# The impact of missing data on analyses of a time-dependent exposure in a longitudinal cohort: a simulation study

**DOI:** 10.1186/1742-7622-10-6

**Published:** 2013-08-19

**Authors:** Amalia Karahalios, Laura Baglietto, Katherine J Lee, Dallas R English, John B Carlin, Julie A Simpson

**Affiliations:** 1 Centre for Molecular, Environmental, Genetic, and Analytic Epidemiology, Melbourne School of Population and Global Health, The University of Melbourne, Parkville, Australia; 2 Cancer Epidemiology Centre, Cancer Council Victoria, Melbourne, Australia; 3 Clinical Epidemiology and Biostatistics Unit, Murdoch Childrens Research Institute, Parkville, VIC, Australia; 4 Department of Paediatrics, The University of Melbourne, Parkville, VIC, Australia

**Keywords:** Simulation study, Missing exposure, Multiple imputation, Complete-case analysis, Repeated exposure measurement

## Abstract

**Background:**

Missing data often cause problems in longitudinal cohort studies with repeated follow-up waves. Research in this area has focussed on analyses with missing data in repeated measures of the outcome, from which participants with missing exposure data are typically excluded. We performed a simulation study to compare complete-case analysis with Multiple imputation (MI) for dealing with missing data in an analysis of the association of waist circumference, measured at two waves, and the risk of colorectal cancer (a completely observed outcome).

**Methods:**

We generated 1,000 datasets of 41,476 individuals with values of waist circumference at waves 1 and 2 and times to the events of colorectal cancer and death to resemble the distributions of the data from the Melbourne Collaborative Cohort Study. Three proportions of missing data (15, 30 and 50%) were imposed on waist circumference at wave 2 using three missing data mechanisms: Missing Completely at Random (MCAR), and a realistic and a more extreme covariate-dependent Missing at Random (MAR) scenarios. We assessed the impact of missing data on two epidemiological analyses: 1) the association between change in waist circumference between waves 1 and 2 and the risk of colorectal cancer, adjusted for waist circumference at wave 1; and 2) the association between waist circumference at wave 2 and the risk of colorectal cancer, not adjusted for waist circumference at wave 1.

**Results:**

We observed very little bias for complete-case analysis or MI under all missing data scenarios, and the resulting coverage of interval estimates was near the nominal 95% level. MI showed gains in precision when waist circumference was included as a strong auxiliary variable in the imputation model.

**Conclusions:**

This simulation study, based on data from a longitudinal cohort study, demonstrates that there is little gain in performing MI compared to a complete-case analysis in the presence of up to 50% missing data for the exposure of interest when the data are MCAR, or missing dependent on covariates. MI will result in some gain in precision if a strong auxiliary variable that is not in the analysis model is included in the imputation model.

## Background

An increasing number of cohort studies are conducting repeated waves of follow-up in order to update information on their participants. This collection of data allows researchers to assess the association between change in an exposure variable, measured prospectively, and the risk of a given outcome variable. However, as with all epidemiological analyses, longitudinal follow-up studies are plagued by the problem of missing data. A recent review of cohort studies that analysed the association between a change in a prospectively measured exposure and an outcome variable found the majority of studies had at least 10% of participants with some missing data; the amount of missing data ranged from 4 to 80% with a median of 25% [1]. Similarly to epidemiological studies with only one wave of data collection, studies with repeated waves of data collection typically exclude participants with any missing data from the epidemiological analysis [1,2]. Excluding participants with missing information, commonly termed a complete-case analysis, reduces the precision of the estimate of the exposure-outcome association and might bias the estimate if the participants with missing data are not similar to those with complete data [3-6]. Multiple imputation (MI) is an alternative method for handling missing data, which has become increasingly accessible to researchers in a number of statistical software packages (e.g. SAS [7] and Stata [8]).

In MI, imputed values for the missing data are generated multiple times resulting in multiple ‘completed’ (observed plus imputed) versions of the dataset; imputed values are sampled from an imputation model that accounts for the uncertainty introduced by the missing data. Each of the completed datasets is then analysed using an appropriate statistical model for the epidemiological analysis and the resulting estimates are combined to produce one overall MI estimate, with a standard error that accounts appropriately for both between- and within-completed data variability of the estimates [9].

Simulation studies present an opportunity to compare the performance of alternative statistical methods to handle missing data since the true values of the parameters being estimated are known. The statistical methods can be evaluated and compared for different amounts of missing data and different missing data scenarios. Increasingly, researchers are basing their simulation studies on data generation models designed to mimic actual epidemiologic studies [3,10-16]. Simulation studies evaluating methods to handle missing covariate data (i.e. missing data in the main exposure variable or confounder variable(s)) have focussed on covariate data measured once for each participant in the study [3,10-13,15,16]. Research investigating methods to handle missing data in repeated waves of data collection have focussed specifically on missing data in repeated measures of the outcome; these methods include extensions of weighted estimating equations, weighted least squares and random effects [14,17,18]. These proposed methods exclude the participants with missing exposure data in the analysis of the exposure-outcome association. Our focus is on how to handle longitudinal data where the repeated exposure measures are subject to missing values but the outcome is completely observed for all participants. To our knowledge this has not been investigated in an extensive simulation study.

The study reported in this paper is based on the Melbourne Collaborative Cohort Study [19] and was motivated by our interest in estimating the magnitude of the association between change in waist circumference, a numerical exposure derived from two measurements of waist circumference, and risk of colorectal cancer, a fully observed time-to-event outcome.

Waist circumference is an established marker for measuring obesity and the association between waist circumference measured at a single point in time and the risk of colorectal cancer is well established [20-25]. Estimating the magnitude of the association between change in waist circumference, and colorectal cancer is important to establish whether the long-term effects of fat loss decrease the risk of colorectal cancer. In the Melbourne Collaborative Cohort Study, waist circumference was measured at baseline and at follow-up, 13 years later, and as with many other cohorts, a significant proportion of participants have missing data at the follow-up assessment and the true missing data mechanism is not known.

The primary aim of this paper was to determine if there was any gain in using MI compared to complete-case analysis for handling up to 50% missing data in a repeatedly measured exposure of interest with a fully observed outcome. Our secondary aim was to assess the impact of including a strong auxiliary variable in MI (i.e. a variable included in the imputation model that is highly correlated with the variable with missing data and is not included in the analysis model). We report the results of a simulation study that compares the performance of MI and complete-case analysis for handling missing data in waist circumference when estimating two associations: change in waist circumference and the incidence of colorectal cancer, and waist circumference at wave 2 and colorectal cancer, with data simulated using two Hazard Ratios (HRs) representing a weak and strong association between change in waist circumference and colorectal cancer, where there were different amounts of missing data according to various missing data mechanisms. Note, throughout this paper the epidemiological analysis of ‘change in waist circumference’ refers to an analysis where the exposure of interest is the absolute change in waist circumference adjusted for waist circumference at baseline.

## Methods

### Motivating example: Melbourne Collaborative Cohort Study

The Melbourne Collaborative Cohort Study is a prospective cohort study of 41,514 people (24,469 women), recruited in Melbourne, Australia, aged between 40 and 69 years at baseline (wave 1). Details of the Melbourne Collaborative Cohort Study have been published elsewhere [19]. In brief, participants were recruited between 1990 and 1994 and extensive baseline demographic, lifestyle (e.g. smoking status, alcohol consumption and physical activity) and dietary information were collected. Anthropometric measures (height, weight, waist and hip circumference) were directly measured by nurses according to written protocols based on standard procedures [26]. A second active wave of follow-up (wave 2) was conducted between 2003 and 2007 in which wave 1 information was updated and anthropometric measures were reassessed. Table 1 provides details of the information collected at each wave. Waist circumference was measured for 99% (41,476) of participants at wave 1 and for 60% (26,846) of participants alive at wave 2. The vital status of the Melbourne Collaborative Cohort Study participants was determined by record linkage to the National Death Index. Colorectal cancer cases were ascertained by record linkage to the population-based Victorian Cancer Registry and to the Australian Cancer Database to identify cases diagnosed in other states of Australia.

**Table 1:** Data structure for the Melbourne Collaborative Cohort Study dataset

**Covariate**	**Variable type**	**Grouping/measurement**	**Label**	**Correlation with **${\mathrm{WC}}_{1}^{*}$
Waist circumference wave 1	Continuous	Centimetres	WC_1_	1.00
Waist circumference wave 2	Continuous	Centimetres	WC_2_	0.81
Age	Continuous	Years	Age	0.18
Sex	Categorical	0 = Males	-	-0.52
		1 = Females	Female	-
Education	Categorical	0 = None or primary school	-	-0.18
		1 = Secondary or trade school	Education _secondary_	-
		2 = Tertiary education	Education _tertiary_	-
Country of birth	Categorical	0 = Australia/New Zealand	-	0.19
		1 = United Kingdom	COB _UK_	-
		2 = Mediterranean	COB _Mediterranean_	-
Smoking status	Categorical	0 = Never smoked	-	0.17
		1 = Former smoker	Smoke _former_	-
		2 = Current smoker	Smoke _current_	
Physical activity score	Categorical	0 = 0	-	-0.13
		1 = (0 to 4)	Physical _low_	-
		2 = [4 to 6)	Physical _moderate_	-
		3 = 6+	Physical _high_	-
Alcohol consumption	Categorical	0 grams (Male & Female)	-	0.05
		1-39 grams (Male) /	Alcohol _low_	-
		1-19 grams (Female)		
		40-59 grams (Male) /	Alcohol _moderate_	-
		20-39 grams (Female)		
		60+ grams (Male) /	Alcohol _high_	-
		40+ grams (Female)		

^*^Correlation with waist circumference at wave 1 was obtained from the observed data of the Melbourne Collaborative Cohort Study participants who attended both waves (i.e. 26,846 participants).

Notwithstanding the limitations of correlations for categorical variables.

### Epidemiological analysis

Two Cox proportional hazards models were fitted to each of the simulated datasets. For both models, age was the time metric and follow-up began at wave 2 and continued until diagnosis of colorectal cancer, date of death or 30 June 2011, whichever came first. The first model (analysis (a)) included absolute change in waist circumference as the covariate of interest (i.e. wave 2 minus wave 1 waist circumference, rescaled so that the estimated HR corresponded to a difference of 10 cm in change in waist circumference), with adjustment for waist circumference at wave 1 as well as for sex, country of birth and education (Equation 1). The second model (analysis (b)) included waist circumference at wave 2 as the exposure of interest, also rescaled so that the estimated HR corresponded to an increase of 10cm in waist circumference at wave 2, with adjustment for sex, country of birth and education (Equation 2).

(1) $$\begin{array}{ll} \log(h_{a}(t)) & =\;\log(h_{a0}(t))+\beta_{a1}({\text{WC}}_{2}-{\text{WC}}_{1})+\beta_{a2}{\text{WC}}_{1}\\ & \;+\beta_{a3}\text{Female}+\beta_{a4}{\text{COB}}_{\text{UK}}+\beta_{a5}{\text{COB}}_{\text{Mediterranean}}\\ & \;+\beta_{a6}{\text{Education}}_{\text{Secondary}}+\beta_{a7}{\text{Education}}_{\text{Tertiary}} \end{array}$$

(2) $$\begin{array}{ll} \log(h_{b}(t)) & =\;\log(h_{b0}(t))+\beta_{b1}{\text{WC}}_{2}+\beta_{b2}\text{Female}+\beta_{b3}{\text{COB}}_{\text{UK}}\\ & \;+\beta_{b4}{\text{COB}}_{\text{Mediterranean}}+\beta_{b5}{\text{Education}}_{\text{Secondary}}\\ & \;+\beta_{b6}{\text{Education}}_{\text{Tertiary}}; \end{array}$$

where t is the time, *β*_*ai*_ and *β*_*bi*_ are the vectors of regression coefficients, *h*_*a*_(*t*) and *h*_*b*_(*t*) are the hazard functions, and *h*_*a*0_(*t*) and *h*_*b*0_(*t*) are the baseline hazard functions, i.e. the hazard function for x = 0, for analysis (a) and (b), respectively.

In the second model there was no adjustment for waist circumference at wave 1, enabling us to assess the impact of an auxiliary variable (i.e. a variable included in the imputation model but not included in the epidemiological analysis) that has a strong association with the variable with missing data (*ρ* = 0.81, Table 1).

### Generating the datasets

This simulation study was based on the 41,476 Melbourne Collaborative Cohort Study participants who had their waist circumference measured at wave 1. We used the observed demographic (i.e. age, sex, country of birth and education) and lifestyle information (i.e. smoking status, physical activity and alcohol consumption) for each participant (see Table 1 for a description of the variables), and simulated data for waist circumference at waves 1 and 2, the outcome of colorectal cancer and the censoring variable of death using relationships from the observed data as described below. We simulated the waist circumference data at waves 1 and 2 to preserve the correlation between the waist circumference measurements at the two waves of data collection conditional on the fixed demographic covariates at baseline. We repeated the simulation process 1,000 times, which allowed us to estimate the log of the HR for change in waist circumference and colorectal cancer to within 1.5% accuracy of the true value [27]. Independent random samples were generated using different starting seeds that were separated by 41,500 units (i.e. a value larger than the sample size) [27]. This produced 1,000 complete simulated datasets of 41,476 individuals for each HR, differing only in terms of waist circumference (at waves 1 and 2), death and the outcome of colorectal cancer.

To preserve the correlations of the exposure variables and the covariates, we simulated waist circumference at waves 1 and 2 from a multivariate normal distribution conditional on continuous (i.e. age) and categorical covariates (i.e. the demographic variables: sex, country of birth, education; and lifestyle factors: smoking status, physical activity and alcohol consumption) [28]. The means and variance-covariance matrix used for the multivariate normal distribution were obtained from the observed data of the 26,846 participants who attended both waves.

Once waist circumference was simulated, we also simulated time to the outcome of colorectal cancer and time to death (i.e. censoring) using two Weibull models [29] with the linear predictors defined in equations (3) and (4) below for colorectal cancer and death respectively based on the analysis of the Melbourne Collaborative Cohort Study data; where ***α***^***′***^ and ***δ***^***′***^ are the vectors of coefficients relating to the vector of covariates **X**:

For time to colorectal cancer:

(3) $$\begin{array}{ll} \alpha^{\prime }X_{\text{colorectal}} & =\;\alpha_{1}\left({\text{WC}}_{2}-{\text{WC}}_{1}\right)+\alpha_{2}{\text{WC}}_{1}+\alpha_{3}\text{Female}\\ & \;+\alpha_{4}\text{Age}+\alpha_{5}{\text{Education}}_{\text{Secondary}}\\ & \;+\alpha_{6}{\text{Education}}_{\text{Tertiary}}+\alpha_{7}{\text{COB}}_{\text{UK}}\\ & \;+\alpha_{8}{\text{COB}}_{\text{Mediterranean}} \end{array}$$

For time to death:

(4) $$\delta^{\prime }X_{\text{death}}=\delta_{1}\text{Female}+\delta_{2}\text{Age}$$

We arbitrarily chose to set the HR associated with a 10cm change in waist circumference to either 1.1 or 1.5, representing a weak and a strong relationship, respectively, with the outcome of colorectal cancer, adjusted for waist circumference at wave 1. The HRs for the remaining covariates, as well as the scale and shape parameters for the Weibull models were set to the values obtained from fitting a Weibull model to the complete Melbourne Collaborative Cohort Study dataset, with time on study set as the time metric.

Once time to event was simulated, participants were classified as either having a colorectal cancer event, or were censored at date of death or end of study (30 June 2011), whichever came first. The date of attendance at wave 2 is required for the epidemiological analysis as this represents the start of follow-up (see Section Epidemiological analysis) and was set to the actual date of attendance for the participants who attended wave 2 and to 30 May 2005 (median date of attendance) for those who did not attend.

### Introduction of missing data

For each of the 1,000 simulated datasets simulated under each “true” HR of 1.1 and 1.5 (i.e. a total of 2,000 datasets) we assigned 15, 30 and 50% of the waist circumference data at wave 2 to missing. These values were chosen to bracket the actual proportion of Melbourne Collaborative Cohort Study participants who did not have their waist circumference measured at wave 2, which was 30%. The waist circumference data were set to missing according to three different scenarios: MCAR and two missing at random scenarios, in both of which missingness is dependent on the covariates, and will be referred to by Little’s [30] terminology ‘covariate-dependent MAR’. For data to be MCAR we selected a random sample of the desired proportion and set their waist circumference at wave 2 to missing.

To generate data according to a covariate-dependent MAR scenario (i.e. such that the distribution of the missingness indicator can be explained by observed variables in the dataset, independently of the missing values themselves), we assumed that the probability of a value being missing followed a logistic regression model (Equation 5) that included the covariates and corresponding parameters defined in Table 2. The predictors of missingness selected for the covariate-dependent MAR scenarios were those observed in the Melbourne Collaborative Cohort Study to be predictors of non-attendance at wave 2. The intercept for the logistic regression model, *γ*_0_ in (5) below, was determined (by iteration) so that the number of observations with missing data was approximately 15, 30 or 50%. In the first covariate-dependent MAR scenario (standard covariate-dependent MAR) we aimed to reflect the missingness pattern observed in the Melbourne Collaborative Cohort Study data using the Odds Ratios (ORs) shown in Table 2. For the second covariate-dependent MAR scenario (enhanced covariate-dependent MAR scenario), we aimed to create a more extreme but still realistic scenario by doubling the log of the ORs used in the first covariate-dependent MAR scenario (equivalent to squaring the ORs).

(5) $$\begin{array}{ll} \text{logit Pr(missing)} & =\;\gamma_{0}+\gamma_{1}{\text{WC}}_{1}+\gamma_{2}\text{Age}+\gamma_{3}\text{Female}\\ & \;+\gamma_{4}{\text{Education}}_{\text{secondary}}+\gamma_{5}{\text{Education}}_{\text{tertiary}}\\ & \;+\gamma_{6}{\text{COB}}_{\text{UK}}+\gamma_{7}{\text{COB}}_{\text{Mediterranean}}\\ & \;+\gamma_{8}{\text{Alcohol}}_{\text{low}}+\gamma_{9}{\text{Alcohol}}_{\text{moderate}}\\ & \;+\gamma_{10}{\text{Alcohol}}_{\text{high}}+\gamma_{11}{\text{Smoking}}_{\text{former}}\\ & \;+\gamma_{12}{\text{Smoking}}_{\text{current}}+\gamma_{13}{\text{Physical}}_{\text{low}}\\ & \;+\gamma_{14}{\text{Physical}}_{\text{moderate}}+\gamma_{15}{\text{Physical}}_{\text{high}} \end{array}$$

**Table 2:** Specification of the logistic regression models used to impose missing data under the two covariate-dependent MAR scenarios

	**Odds ratio for missing (exp(*γ***_***i***_**))**	
*γ*	Scenario 1	Scenario 2 ^$^
1 (WC_1_, 10 cm)	1.10	1.21
2 (Age, years)	1.06	1.12
3 (Female)	1.10	1.21
4 (Education _secondary_)	0.72	0.52
5 (Education _tertiary_)	0.44	0.19
6 (COB _UK_)	1.15	1.32
7 (COB _Mediterranean_)	1.71	2.92
8 (Alcohol _low_)	0.77	0.59
9 (Alcohol _moderate_)	0.66	0.44
10 (Alcohol _high_)	0.85	0.72
11 (Smoking _former_)	1.16	1.35
12 (Smoking _current_)	1.80	3.24
13 (Physical _low_)	0.93	0.86
14 (Physical _moderate_)	0.99	0.98
15 (Physical _high_)	0.91	0.83

^$^Odds ratio for Scenario2 = (Odds ratio for Scenario1) ^2^.

### Methods to handle missing data

We compared two methods to handle missing data: complete-case analysis and MI. In the complete-case analysis the HRs were estimated in each of the 1,000 datasets after excluding individuals with a missing waist circumference at wave 2. For MI, 20 sets of imputed values were generated for the missing data in each of the 1,000 datasets, by sampling from a Gaussian normal regression model (using Stata’s ‘mi impute regress’ command). The imputation model included the covariates used to generate the covariate-dependent MAR missing data (i.e. the covariates that were included in the Cox regression, which ensures the maximum recovery of information about the associations of interest [31]), as well as additional auxiliary variables measured at wave 1, an indicator for whether a participant had colorectal cancer or was censored at the time of analysis, and the baseline hazard generated using the Nelson-Aalen method [3,32]. All categorical covariates included in the imputation model were represented as k-1 indicator variables (where k represents the number of categories). Cox proportional hazards models were then fitted separately to each of the 20 ‘completed’ datasets, and the resulting estimates (log(HR)) of the effect of interest were averaged to produce an overall MI estimate with a corresponding standard error calculated using Rubin’s rules [33,34].

### Evaluation of biases, precision and coverage

We focussed on the estimation of the exposure-outcome relationship of primary interest, the HR for change in waist circumference and colorectal cancer (analysis (a)) and for waist circumference at wave 2 and colorectal cancer (analysis (b)). For analysis (a), the true value for log(HR) associated with the exposure of interest was the value that was used in Equation (1) (i.e. log(1.1) and log(1.5)). However, analysis (b) fits a different model than that under which the data were simulated (not adjusted for waist circumference at wave 1), and therefore, the “complete” value of the log(HR) was taken to be that obtained by running the Cox proportional hazard model on 10,000 simulated datasets, before any values were assigned to missing. This gave “complete” values of log(1.14) and log(1.54). In both cases, the average of the standard errors of the estimated log(HR) from 10,000 simulated datasets prior to assigning any data to missing was considered the “complete” standard error. We compared the performance of the complete-case analysis with MI for handling missing data using: absolute bias of the log(HR), the difference between the true/complete value and the average of the estimated log(HR) calculated for each of the 1,000 simulated datasets; the empirical standard error, the average standard deviation of the estimates of interest from 1,000 simulations; and the coverage, the percentage of the 95% confidence intervals that included the true value [27].

The data were simulated and the statistical analyses were performed using Stata version 11.2 [8].

## Results

**Figure 1:**
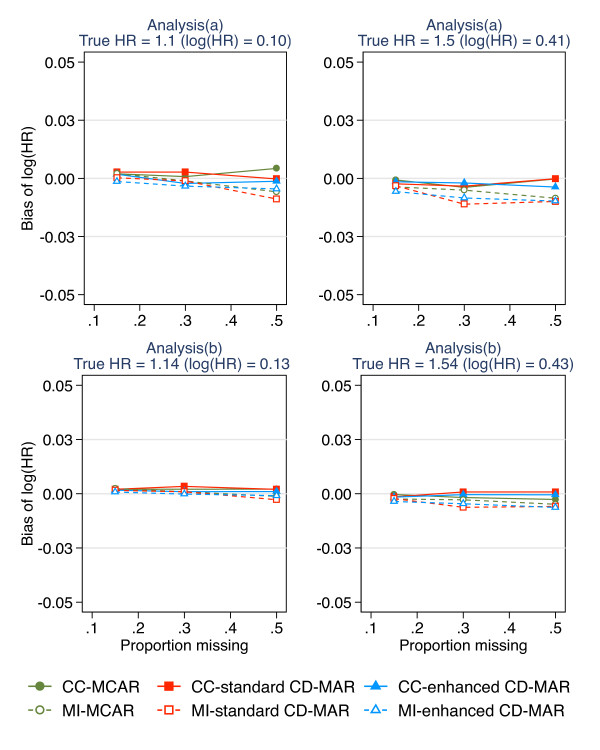
Absolute bias for complete-case analysis and MI for increasing proportions of missing data (0.15, 0.3, 0.5) under three missing data scenarios (a) corresponds to the epidemiological analysis of absolute change in waist circumference adjusting for waist circumference at wave 1; (b) corresponds to the epidemiological analysis of waist circumference at wave 2 (without adjusting for waist circumference at wave 1); CD-MAR refers to Covariate-dependent MAR scenario.

With increasing proportions of missing data in waist circumference at wave 2 there was no bias using complete-case analysis under any missing data scenario (i.e. MCAR, standard covariate-dependent MAR or enhanced covariate-dependent MAR) for analysis (a), whereas slight bias was observed using MI to handle the missing data. However, even with up to 50% missing data the bias was minimal and did not exceed 0.01 (absolute change in log(HR)). The bias observed under analysis model (b) was negligible using both complete-case analysis and MI to handle the missing data and did not exceed 0.005 (Figure 1). The Monte Carlo error, i.e. the noise from the finite number of simulations, did not exceed 0.4%.

**Figure 2:**
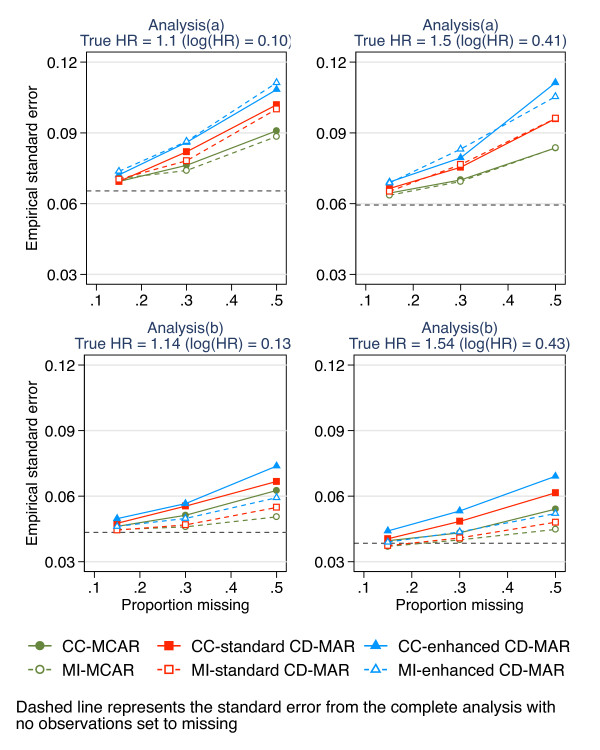
Empirical standard error for complete-case analysis and MI for increasing proportions of missing data (0.15, 0.3, 0.5) under three missing data scenarios (a) corresponds to the epidemiological analysis of absolute change in waist circumference adjusting for waist circumference at wave 1; (b) corresponds to the epidemiological analysis of waist circumference at wave 2 (without adjusting for waist circumference at wave 1); CD-MAR refers to Covariate-dependent MAR scenario.

Figure 2 shows the empirical standard errors of the exposure-outcome estimate. The empirical standard errors are, as expected, greater than the “complete” standard error for all proportions of missing data and increase with increasing proportions of missing data using the complete-case analysis and MI. For analysis (a), MI showed minimal, if any, gain in precision for up to 50% missing data compared to complete-case analysis. There were considerably larger gains from MI with analysis (b), which included the strong auxiliary variable, waist circumference at wave 1, in the imputation model (Figure 2).

**Figure 3:**
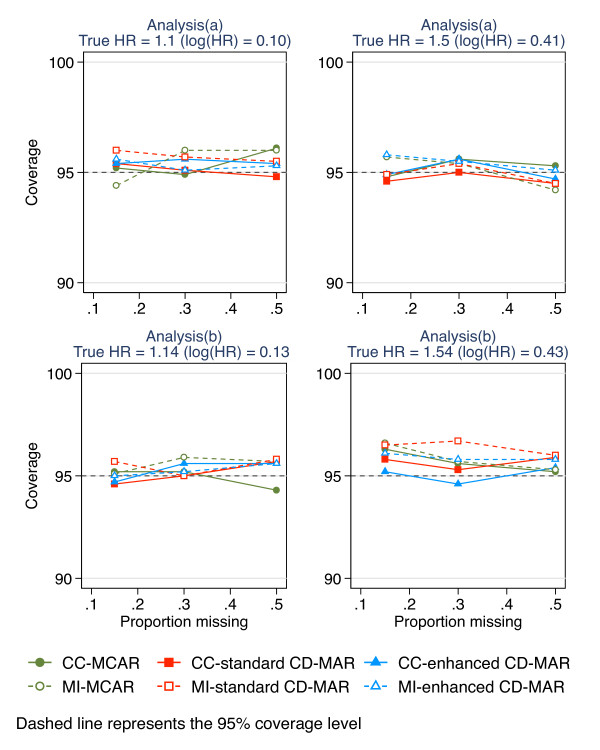
Coverage of the regression coefficient estimates for complete-case analysis and MI for increasing proportions of missing data (0.15, 0.3, 0.5) under three missing data scenarios (a) corresponds to the epidemiological analysis of change in waist circumference adjusting for waist circumference at wave 1; (b) corresponds to the epidemiological analysis of waist circumference at wave 2 (without adjusting for waist circumference at wave 1); CD-MAR refers to Covariate-dependent MAR scenario.

The coverage for the different missing data methods in relation to an increasing proportion of missing data is shown in Figure 3. The coverage of the true values for analysis models (a) and (b) remained near the nominal 95% level with up to 50% missing data for the two methods used to handle the missing data.

## Discussion

Using a simulation study based on the Melbourne Collaborative Cohort Study we assessed two methods (i.e. complete-case analysis and MI) for handling up to 50% missing data in a repeatedly measured exposure of interest, in the context of a Cox proportional hazards model investigating two epidemiological associations. We found very little bias and the coverage remained around 95% from both complete-case analysis and MI for both associations. For the analysis that included absolute change in waist circumference as the exposure of interest and adjusted for waist circumference at wave 1 (i.e. analysis (a)) there was no gain in precision when using MI instead of a complete-case analysis. However, there were slight gains in precision (i.e. reduction in the standard error) for MI over a complete-case analysis in analysis (b), where there was a strong auxiliary variable in the imputation model.

The simulation study that we developed was based on a large existing cohort study, the Melbourne Collaborative Cohort Study. This approach for designing simulation studies (i.e. based on real studies), which is becoming increasingly common in the literature, allows researchers to incorporate complex and realistic associations within the data structure while simplifying the data generation process compared to a fully simulated scenario [3,10-12,15,35,36]. As with all simulation studies of this type, the generalisability of our findings is limited since our simulated data are based on only a single cohort. Undoubtedly, further exploration of simulation models based on other real data settings would be useful. As well, there is scope to investigate datasets with more than two waves of data collection to ascertain whether there is any gain in using MI compared to complete-case analysis in these scenarios.

The true HRs that we chose for the association between a 10cm change in waist circumference and risk of colorectal cancer (i.e. 1.1 and 1.5) were based on realistic HRs that are typically observed for this anthropometric measure [25,37,38]. These HRs of moderate magnitude may have minimised the bias that we observed but our results are consistent with previous work that investigated HRs of similar magnitude; Demissie et al. [10] found little bias for a HR of one for a dichotomous exposure variable and Marshall et al. [3] found minimal bias for an exposure with a HR of one for a unit change in a continuous exposure variable.

We imposed our missing data on the exposure of interest under MCAR, standard and enhanced covariate-dependent MAR scenarios. Consistent with previously published studies, we found that under the MCAR scenario complete-case analysis produced negligible bias and good coverage of the estimate [3,10,11]. The covariate-dependent MAR scenarios that we investigated were based on the variables observed to be predictors of non-attendance at wave 2 in the Melbourne Collaborative Cohort Study (i.e. missing waist circumference at wave 2) and the coefficients for the standard covariate-dependent MAR scenario were set to the same values as the estimates of the regression coefficients from the logistic regression model of missingness indicator at wave 2. This realistic missing data scenario allowed us to evaluate the two methods for handling missing data under weak associations of covariate-dependent MAR, which are more likely to be observed in real studies than the more extreme covariate-dependent MAR scenarios that are often reported in the missing data literature [11,12,39,40]; for example, Donders et al. [39] assigned 40% of the simulated data to missing with all of the missing data occurring in the unexposed group.

We found very little bias in the log(HR) using complete-case analysis and MI to handle the missing data. The slight bias observed in analysis (a) may be a result of the imputation model being semi-compatible with the analysis model (i.e. the exposure of interest in the analysis model is change in waist circumference, however, waist circumference at wave 2 is imputed in the imputation model) [41]. We decided to impute waist circumference at wave 2 instead of change in waist circumference in order to represent the real epidemiological analysis (i.e. in a study where the variable is fully observed at wave 1 and missing at wave 2, the analyst is more likely to impute the variable with missing data and then calculate the absolute change between the two variables). The imputation model that we used included an indicator variable for whether a participant had colorectal cancer or was censored at the time of analysis, and the baseline hazard generated using the Nelson-Aalen method in the imputation model. Although Marshall et al. [3] suggest that this may be a better method to use in the imputation model than including a log transformation of the survival time and event status it may have introduced bias into our results [32]. Further, our MAR scenario was a covariate-dependent scenario, which may be specific to our study and research looking at MAR scenarios dependent on both covariates and the outcome should be considered. Previous published simulation studies, which reported biased estimates using complete-case analysis or MI, induced a missingness mechanism dependent on the exposure and outcome variables and under more extreme missingness scenarios [3,10,11,15,40].

The auxiliary variables included in our imputation model (i.e. variables not included in the epidemiological analyses) were alcohol intake, smoking status, and physical activity at baseline. These variables had only weak to moderate associations with waist circumference at wave 2. To assess the impact of an auxiliary variable that has a strong association with the exposure of interest we compared MI with a complete-case analysis for handling missing data under two scenarios: (a) the association between change in waist circumference and risk of colorectal cancer adjusted for waist circumference at wave 1, and (b) waist circumference at wave 2 and the risk of colorectal cancer, not adjusted for waist circumference at wave 1. For analysis (b), waist circumference at wave 1 was included in the imputation model as a strong auxiliary variable, with no strong auxiliary variables in model (a). MI provided no gain in precision of the estimate compared to complete-case analysis in analysis (a) where the imputation procedure only included auxiliary variables with weak and moderate associations with the variable with missing data. However, slight gains in precision were observed for the MI estimate compared to the complete-case estimate in analysis (b). Graham and Collins [42] used simulations of artificial data to show that strong auxiliary variables included in the imputation model for MI restored some of the power lost due to missing data. Real data examples are less likely to have auxiliary variables that are strongly associated with the variable subject to missing values; for example Marshall et al. [3] reported correlations in the range of 0.3 and 0.4, and Lee and Carlin [43] reported correlations between 0.1 and 0.6 between the covariates and the variable with missing data. Incorporating auxiliary variables with weak or moderate associations with the variables with missing data into the imputation model will result in large between-imputation variance leading to larger standard errors for the MI estimates and thus, smaller gains (if any) from using MI compared to a complete-case analysis.

Complete-case analysis is the default method for most software packages for handling missing data in statistical analyses. However, MI is now available and easy to implement in many software packages (e.g. Stata, R, SAS and SPSS [7,8,44,45]). This increased accessibility has led to an increase in the use of MI for dealing with missing data in epidemiological studies [34,46]. MI produces unbiased estimates if the missing data mechanism is MAR, which encompasses the more specific scenario of covariate-dependent MAR [4,47]. Data Missing Not at Random (MNAR) occur when the study participants with missing data differ from the study participants with complete data in a manner that cannot be explained by the observed data [9]. In our simulation study we did not include an MNAR missing data scenario. It has been suggested that for cohort studies that collect a large amount of information from their participants, the observed data can provide a lot of information about the missing data. Further, the imputation model may include combinations of observed variables that represent surrogate measures of the unobserved variables that are related to the missingness mechanism [48]. However, whether the data are MAR, either covariate-dependent or more generally, is untestable and therefore, further research investigating alternative approaches that explore the sensitivity of conclusions to plausible MNAR mechanisms or simultaneously estimate the missing data model and the analysis model will be important [49].

## Conclusion

The findings from this simulation study, which used a data generation model designed to replicate a large longitudinal cohort study, demonstrate that for an epidemiological study assessing the association between change in an exposure measure assessed at two waves of data collection, with up to 50% missing data at the second measurement, and a fully observed time to event outcome, there is no advantage in using MI over the simple complete-case analysis approach when the missing data are only associated with other measured covariates and not the outcome. MI did show gains in precision for an analysis that ignored a strongly predictive baseline measure, which became a strong auxiliary variable when included in the imputation model. However, the latter example was somewhat artificial and it seems unlikely that in many real data scenarios there will be auxiliary variables (i.e. variables not already included as covariates in the analysis model) that exhibit such strong associations with the variable subject to missing data.

## Abbreviations

MCAR: Missing completely at random; MAR: Missing at random; MNAR: Missing not at random; MI: Multiple imputation; HR: Hazard ratio.

## Competing interests

The authors declare that they have no competing interests.

## Authors’ contributions

AK designed the simulation study, performed the analysis and drafted the manuscript. LB and KJL provided assistance in setting up the simulation study and provided feedback on the manuscript. DRE and JBC provided feedback on the design of the simulation study and drafts of the manuscript. JAS conceived of the idea for the simulation study, participated in designing the simulation study and helped in writing of the manuscript. All authors have read and approved the final manuscript.
